# Paricalcitol alleviates intestinal ischemia-reperfusion injury via inhibition of the ATF4-CHOP pathway

**DOI:** 10.3389/fphar.2025.1529343

**Published:** 2025-04-03

**Authors:** Jiawei Zhang, Tingting Liu, Tongqing Xue, Zhongzhi Jia

**Affiliations:** ^1^ Department of Interventional and Vascular Surgery, The Third Affiliated Hospital of Nanjing Medical University (Changzhou Second People’s Hospital), Changzhou, China; ^2^ Graduate College of Dalian Medical University, Dalian, China; ^3^ Department of Interventional Radiology, Huaian Hospital of Huai’an City (Huaian Cancer Hospital), Huai’an, China

**Keywords:** intestinal, ischemia reperfusion injury, paricalcitol, ATF4, CHOP, VDR (vitamin D receptor)

## Abstract

**Introduction:**

Intestinal ischemia reperfusion (I/R) injury is a severe condition characterized by inflammation, oxidative stress, and compromised intestinal barrier function, which can lead to death. This study investigated the effects of paricalcitol, a synthetic vitamin D receptor (VDR) agonist, on intestinal I/R injury, focusing on the activating transcription factor 4 (ATF4)-C/EBP homologous protein (CHOP) signaling pathway and the modulation of endoplasmic reticulum stress (ERS).

**Methods:**

This study consists of both *in vivo* and *in vitro* experiments. *In vivo* experiment, a mouse model of intestinal I/R injury was established by clamping the superior mesenteric artery, and followed by 24 or 72 h of reperfusion. 6-week-old male C57BL/6 J mice were randomly assigned to six groups: sham, I/R 24h, I/R 72 h, and their respective paricalcitol-treated counterparts. VDR knockout mice and wild-type mice were assigned to WT, VDR-KO, WT + I/R and VDR-KO + I/R groups. The paricalcitol-treated groups received oral gavage of paricalcitol (0.3 μg/kg) once daily for 5 days before I/R. *In vitro*, IEC-6 cells were incubated in a microaerophilic system (5% CO_2_, 1% O_2_, 94% N_2_) for 6 h to induce hypoxia. The cells were then transferred to complete medium with or without paricalcitol (200 nM) and cultured under normoxic conditions for 24 h to establish the hypoxia/re-oxygenation (H/R) model and investigate the protective effects of paricalcitol on H/R-induced injury in cells. We further utilized VDR- and ATF4-silenced cells to examine how paricalcitol regulates the expression of VDR, ATF4, and CHOP.

**Results:**

We demonstrated that protective paricalcitol treatment reduces ERS and apoptosis by activating VDR and inhibiting the ATF4-CHOP pathway, thereby alleviating intestinal I/R injury *in vivo* and H/R injury *in vitro*. Furthermore, experiments with VDR knockout mice demonstrated that the absence of VDR exacerbated I/R injury, underscoring the protective role of VDR in intestinal epithelial cells.

**Discussion:**

These findings suggest that the protective effects of paricalcitol may offer a promising therapeutic strategy for managing intestinal I/R injury.

## Introduction

Intestinal ischemia reperfusion (I/R) injury is a life-threatening condition that is typically caused by ischemic or septic shock in patients with acute mesenteric ischemia (Reintam et al., 2024). Intestinal I/R injury is a complex condition marked by the excessive release of inflammatory cytokines and oxidative stress, leading to epithelial cell death and compromised intestinal barrier function. This disruption results in increased intestinal permeability and reduced nutrient absorption, which can facilitate the passage of macromolecules (Wang et al., 2024). Currently, there is no established clinical treatment for intestinal I/R injury.

Recent research has focused on the role of endoplasmic reticulum stress (ERS) and the activating transcription factor 4 (ATF4)-C/EBP homologous protein (CHOP) pathway in the pathogenesis of I/R injury (Tang et al., 2023; Zhao et al., 2024). ERS occurs when there is an excessive accumulation of unfolded or misfolded proteins in the endoplasmic reticulum. Excessive ERS can trigger inflammation and eventually lead to programmed cell death (Stengel et al., 2020). ATF4, a member of the activating transcription factor family, plays a crucial role in gene regulation (Chen et al., 2022). Under normal conditions, its expression is low but significantly upregulated upon stimulation (Gachon et al., 2001). Previous studies have shown that ATF4 plays a key role in apoptosis and regulates various physiological processes, including amino acid metabolism, redox homeostasis, and mitochondrial function (Wei et al., 2021; Ohoka et al., 2005). As a key effector of ERS, ATF4 regulates downstream genes involved in apoptosis, inflammation, and oxidative stress (Steiger et al., 2004). CHOP, another critical mediator of ERS-induced cell death, is upregulated by ATF4. Their overexpression enhances oxidative stress and cell death (Han et al., 2013). Notably, inhibition of ATF4-CHOP signaling has been shown to reduce mitophagy, ERS, apoptosis and ischemia-reperfusion injury (Tang et al., 2023; Chen et al., 2022; Chen et al., 2024).

The vitamin D receptor (VDR) is a nuclear transcription factor that is widely present in cells of various tissues and is highly expressed in the intestines (Hamza et al., 2023). Recent research has shown that VDR has immunoregulatory effects, promotes the differentiation and proliferation of intestinal tissues, and plays an important role in maintaining the normal barrier function of intestinal epithelial cells (Sun and Zhang, 2022). Additionally, experimental evidence has suggested a close association between VDR and I/R injury in organs such as the heart, liver, brain, and kidneys (Qian et al., 2019; Wu et al., 2023; Kim et al., 2017). Numerous studies have demonstrated that activating VDR can inhibit ERS (Zhou et al., 2020; Haas et al., 2016; Ahmad et al., 2022), and activation of VDR has been found to alleviate I/R-induced renal injury by suppressing ERS, partly through transcriptional regulation of the ATF4/CHOP pathway (Tang et al., 2023). However, no studies to date have demonstrated whether paricalcitol can mitigate intestinal I/R injury or whether the ATF4-CHOP pathway plays a role in the progression of this injury.

The aim of this study was to evaluate the effects of paricalcitol, a synthetic VDR agonist, in an intestinal I/R model, focusing on the ATF4-CHOP signaling pathway.

## Materials and methods

### Animals

All animals in this study were treated in accordance with the National Institutes of Health Guide for Care and Use of Laboratory Animals. And all procedures were approved by the animal ethics committee of Changzhou Second People’s Hospital (Permit Number: 2024KY206-01).

C57BL/6 mice (6 weeks old, 16–21 g) (Huachuang Sino Tech Ltd., Nanjing, China) and VDR knockout (VDR-KO) mice (6 weeks old, 16–21 g) (Gem Pharma Tech Ltd., Nanjing, China) were used for the *in vivo* studies. Because of the salutary effect of estrogen, female mice are more resistant to intestinal I/R injury than male mice (Chai et al., 2019; Ricardo-da-Silva et al., 2017); therefore, only male mice were used. Animals were housed in ventilated cages at a temperature of 20°C–24°C, with relative humidity (40%–70%) and a 12-h light/dark cycle. The mice had free access to food and water. All of the mice were allowed to adapt to this environment for 1 week before any experiments were conducted.

### Intestinal I/R injury model and experimental groups

The intestinal I/R injury models were established as described previously (Wen et al., 2019; Jia et al., 2017). Briefly, the mice were anesthetized with 2% pentobarbital sodium (0.3 mg/10 g) via intraperitoneal injection. A midline incision was made, and the superior mesenteric artery (SMA) was clamped with a microvascular clip for 45 min to induce ischemia. The clip was then removed and the incision was sutured, and this was followed by 24 or 72 h of reperfusion. Paricalcitol (HY-50919, MCE, Princeton, NJ, United States) and corn oil were administered once daily for five consecutive days before surgery, according to the specific treatment groups.

The C57BL/6 mice were randomly assigned to one of six groups (n = 6 per group): sham or paricalcitol groups, in which mice underwent laparotomy without SMA occlusion and received oral gavages of corn oil (0.5 mL/10 g) or paricalcitol (0.3 μg/kg) once daily for 5 days before the procedure; I/R 24 h or I/R 72 h groups, in which mice underwent 45 min of ischemia followed by 24 or 72 h of reperfusion, and were given corn oil (0.5 mL/10 g) before the procedure; or paricalcitol + I/R 24 h or paricalcitol + I/R 72 h groups, in which mice underwent ischemia and 24 or 72 h of reperfusion, with paricalcitol (0.3 μg/kg) administered once daily for 5 days before the procedure based on data from previous studies (Hong et al., 2017).

The VDR-KO mice were randomly divided into two groups (n = 6 per group): an VDR-KO + I/R group, in which the VDR-KO mice underwent 45 min of ischemia followed by 72 h of reperfusion; and an VDR-KO group, which did not undergo ischemia or reperfusion. Similarly, wild-type (WT) mice were divided into corresponding groups including a WT group and a WT + I/R group, following the same protocol as the VDR-KO mice.

After reperfusion, the mice were euthanized via exsanguination under isoflurane anesthesia, followed by cervical dislocation. Two 0.5-cm segments of the ileum were collected 10 cm proximal to the terminal ileum. One segment was fixed in 10% formalin and embedded in paraffin for histopathological evaluation, while the other was preserved in electron microscope fixative (glutaraldehyde, 4%) for subsequent transmission electron microscopy analysis. Additionally, a 10-cm segment was washed with PBS, dried, and stored at −80°C for further biochemical analysis.

### Biochemical analysis

The intestinal tissues were homogenized in ice-cold normal saline. The homogenates were then centrifuged at 3500 *g* at 4°C for 20 min. The supernatant fraction of the intestinal homogenates was collected, and the levels of superoxide dismutase (SOD) and glutathione (GSH) were determined using ELISA kits (A001-three to two, A006-one to one, Jianchen, Nanjing, China).

### Histological and immunohistochemistry (IHC) analysis

The isolated ileum segments were fixed in 4% paraformaldehyde for at least 24 h and then embedded in paraffin and sectioned into 4-μm-thick slices. The segments were stained with hematoxylin and eosin (H&E) for histological analysis. The H&E-stained images were assessed using a digital pathology scanner (KFBIO, Zhejiang, China) and evaluated at ×5 magnification. The images were scored by three pathologists blinded to this research according to the methods of Chiu et al. (1970). The Chiu scoring criteria are as follows: 0 – normal intestinal mucosal villi morphology; one – expansion of the subepithelial Gruenhagen’s space in the villous core, capillary congestion, and epithelial damage; 2 – further expansion of the subepithelial space with significant separation between the epithelial layer and the lamina propria; three – increased subepithelial space with occasional denuded villous tips; 4 – severe villous damage and denudation, accompanied by capillary dilation in the lamina propria; 5 – ulceration and hemorrhage of the lamina propria.

For IHC analysis, the ileum segments were deparaffinised with xylene and were then hydrated in serial dilutions of alcohol. The sections were immersed in 3% hydrogen peroxide solution to inhibit endogenous peroxidase activity and were then incubated with antibodies against the tight junction protein zonula occludens-1 (ZO-1) (82870-7-RR, Proteintech, Wuhan, China; 1:100) or VDR (12550S, Cell Signaling Technology, Danvers, MA, United States; 1:100). This was followed by the addition of anti-rabbit immunoglobulin and streptavidin conjugated to horseradish peroxidase. The ileum segments were then stained with 3,3′-diaminobenzidine (DAB) and hematoxylin for counter staining. A digital pathology scanner was used to assess the segments. The protein expression levels and histological changes were evaluated at ×10 magnification.

### Transmission electron microscopy

The intestinal tissues were placed in electron microscope fixative (glutaraldehyde, 4%) at 4°C. The tissue was then embedded and cut into ultrathin sections of 60–80 nm. This was followed by uranium-lead double staining. The morphology of the endoplasmic reticulum in the intestinal epithelial cells was observed using transmission electron microscopy.

### Cell culture and hypoxia/re-oxygenation (H/R) model

IEC-6 cells (Pricella, Wuhan, China) were cultured in DMEM with 10% fetal bovine serum. Paricalcitol treatment concentration (200 nM) was determined based on a previous study by Tang et al. (2023). The experiment consisted of three phases. In the first phase, cells were divided into control, paricalcitol, H/R, and H/R + paricalcitol groups. Cells of the HR group were incubated in a microaerophilic system (Thermo Fisher Scientific, Waltham, MA, United States) with 5% CO_2_ and 1% O_2_ and balanced with 94% N_2_ for 6 h, followed by 24 h of reoxygenation, while the H/R + paricalcitol group was reoxygenated in complete medium containing paricalcitol (200 nM) for 24 h. In the second phase, cells were assigned to control, siVDR, siVDR + H/R, and siVDR + H/R + paricalcitol groups. In the siVDR group, cells were transfected with siVDR using Lipofectamine 3000 (L3000015, Invitrogen, Carlsbad, CA, United States), incubated with 50 nM siRNA for 6 h, and then cultured in complete medium with or without paricalcitol (200 nM) for 24 h. Hypoxia and reoxygenation conditions were identical to those in the first phase. In the third phase, siATF4 replaced siVDR, forming control, siATF4, siATF4 + H/R, and siATF4 + H/R + paricalcitol groups, with experimental conditions identical to those in the second phase.

### Cell counting Kit-8 assays

Cell viability was assessed using the Cell Counting Kit-8 (CCK-8) (CK04, Dojindo, Japan) following the manufacturer’s instructions. IEC-6 cells were seeded in 96-well plates (5,000 cells/well), and 10 μL of CCK-8 reagent was added to each well. After 1 h of incubation at 37°C, absorbance was measured at 450 nm. Data were analyzed using GraphPad Prism 5.0 (GraphPad Prism Software, San Diego, CA, United States).

### TUNEL assay

Apoptotic cells were detected using the TUNEL assay kit (E-CK-A320, Elabscience, Wuham, China), following the manufacturer’s instructions. IEC-6 cells were seeded in 24-well plates. After treated, cells were fixed with 4% paraformaldehyde at room temperature for 30 min, then incubated with a terminal deoxynucleotidyl transferase reaction mixture at 37°C for 1 h. After washing with PBS, the cells were counterstained with 4′,6-diamidino-2-phenylindole (DAPI) and observed under a fluorescence microscope.

### Western blotting

Total protein was extracted from the intestinal mouse tissues and from the IEC-6 cells. Proteins were separated on 10% SDS-PAGE gels and were then transferred to PVDF membranes (Immobilon, Darmstadt, Germany). The membranes were blocked with fast blocking buffer (Servicebio, Wuhan, China) and incubated overnight with primary antibodies, including VDR (12550S, Cell Signaling Technology, Danvers, MA, United States), ATF4 (28657-1-AP, Proteintech, Wuhan, China), CHOP (15204-1-AP, Proteintech, Wuhan, China) and β-actin (HRP-81115, Proteintech, Wuhan, China). After three washes, they were incubated with a secondary antibody (SA00001-2, Proteintech, Wuhan, China) for 1 h. Protein detection was performed using an enhanced chemiluminescence (ECL) system, and quantification was performed using ImageJ, normalized to β-actin.

### Statistical analysis

Parametric data with normal distributions were expressed as mean ± standard deviation (SD) and analyzed using one-way analysis of variance (ANOVA) followed by the Student-Newman-Keuls (SNK) test. Non-parametric data were analyzed using the Kruskal–Wallis test, followed by Dunn’s *post hoc* test, and presented as median ± range (minimum–maximum). All experimental results were obtained from at least three independent experiments. Statistical analysis was performed using GraphPad Prism 5.0. Statistical significance was inferred at *P* values <0.05.

## Results

### Paricalcitol alleviated intestinal I/R injury

**FIGURE 1:**
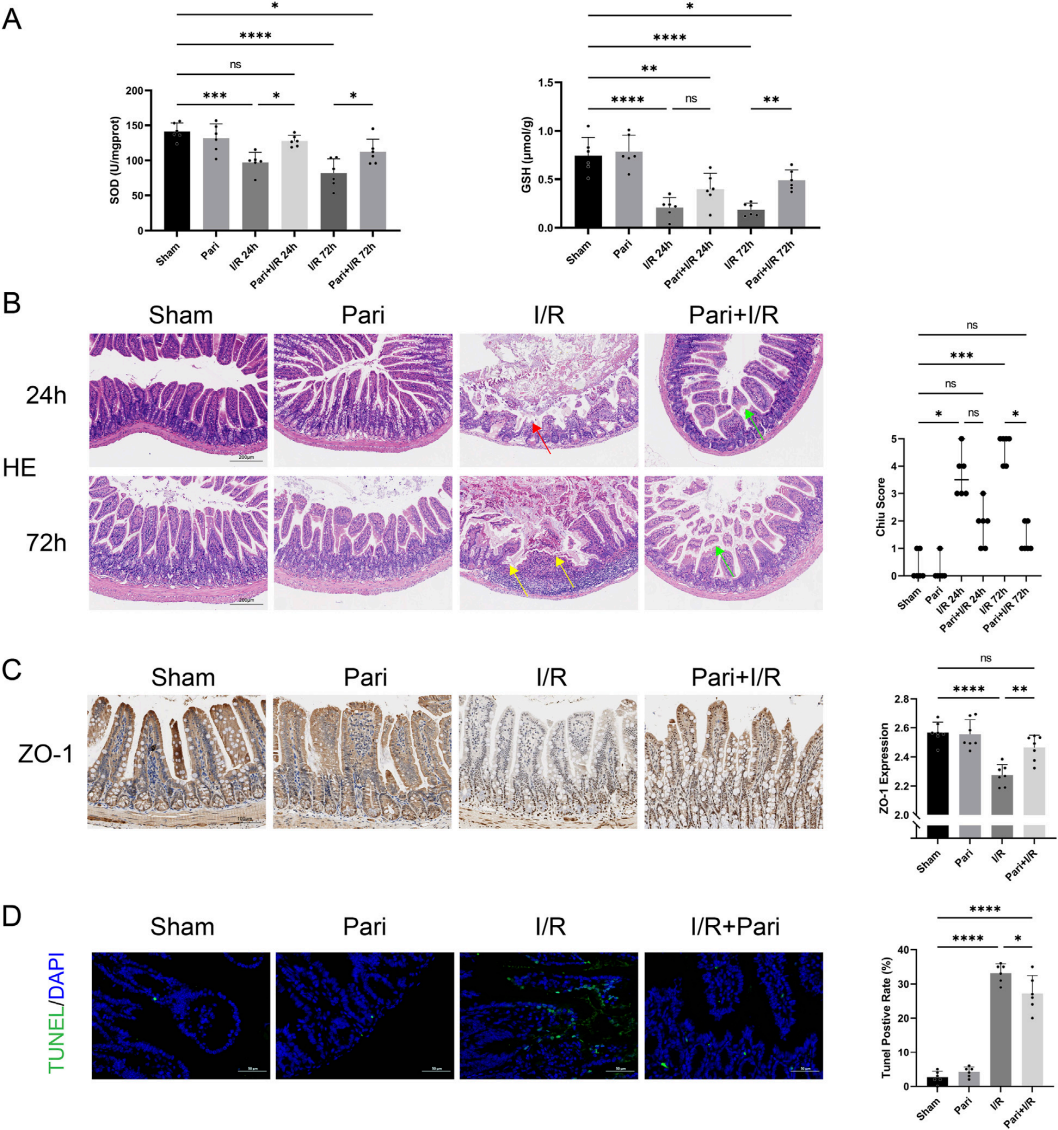
Paricalcitol alleviates intestinal injury and apoptosis caused by ischemia reperfusion (I/R) **(A)** Levels of superoxide dismutase (SOD) and glutathione (GSH) in intestinal tissues across various groups (n = 6 in each group). **(B)** Representative images of intestinal hematoxylin and eosin (H&E) staining results and Chiu scores. Red arrow represents severe villous denudation, yellow arrow represents severe villous denudation and destruction to the lamina propria, green arrow represents mild denudation of the villous tips. (scale bar = 200 μm; n = 6 in each group). **(C)** Representative immunohistochemistry results and expression analysis zonula occludens-1 (ZO-1) in intestinal tissues after 72 h reperfusion (scale bar = 100 μm; n = 6 in each group). **(D)** Representative TUNEL staining (green) and nuclear staining (blue) results and apoptosis analysis of intestinal tissues after 72 h reperfusion (scale bar = 50 μm; n = 6 in each group). Statistical analysis was performed using one-way ANOVA followed by Tukey’s *post hoc* test for parametric data and the Kruskal–Wallis test followed by Dunn’s *post hoc* test for non-parametric data. Parametric data are presented as mean ± SD, while non-parametric data are presented as median (min-max). *, P values <0.05; **, *P* values <0.01; ***, *P* values <0.001; ****, *P* values <0.0001.

In the intestinal I/R injury model, reperfusion for 24h and 72 h reduced average SOD activity to 69% and 57% of the sham group, respectively. However, with paricalcitol pretreatment, SOD activity decreased only to 90% and 79% of the original levels, showing a significant increase compared to untreated mice (P < 0.05). Similarly, paricalcitol pretreatment significantly opposed the I/R-induced reduction in GSH levels, increasing GSH levels by 2.7-fold after 72 h of reperfusion compared to the I/R 72 h group (P < 0.01). Similar results were observed in the assessments of H&E-stained images and Chiu scores. In the I/R 24 h group, severe mucosal epithelial detachment was observed, while in the I/R 72 h group, more severe epithelial necrosis and hemorrhage were noted, leading to an increase in the Chiu scores (P < 0.01). However, pretreatment with paricalcitol significantly alleviated intestinal epithelial injury, with only mild mucosal epithelial detachment. Furthermore, there was no significant difference in the Chiu scores compared to the sham group (Figure 1B). IHC analysis demonstrated that paricalcitol significantly mitigated the loss of ZO-1 caused by I/R injury in intestinal tissue and restored ZO-1 expression to normal levels (Figure 1C). Additionally, TUNEL assay indicated that paricalcitol significantly reduced I/R-induced intestinal cell apoptosis. Compared to the I/R group, paricalcitol pretreatment reduced the apoptosis rate of intestinal epithelial cells by 18% (*P* < 0.05) (Figure 1D).

### Paricalcitol inhibited I/R-induced ERS in intestinal mucosal epithelial cells through the activation of VDR

**FIGURE 2:**
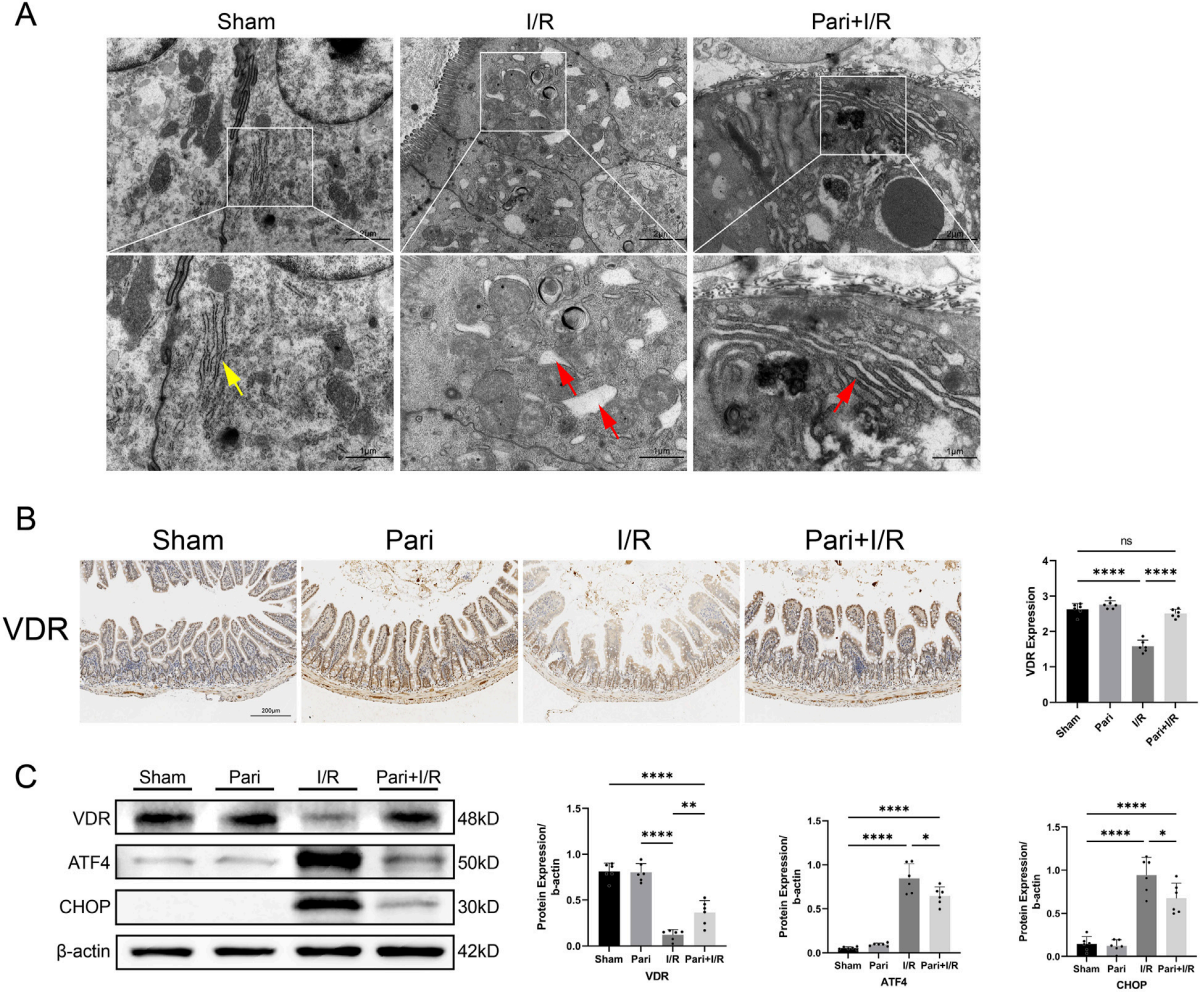
Paricalcitol alleviates endoplasmic reticulum stress induced by intestinal ischemia reperfusion (I/R) injury through the activation of vitamin D receptor (VDR) **(A)** Representative transmission electron microscopy images showing endoplasmic reticulum damage in intestinal epithelial cells. Yellow arrows represent normal endoplasmic reticulum, red arrows represent endoplasmic reticulum changes (scale bar = 2 or 1 μm; n = 3 in each group). **(B)** Representative immunohistochemistry results for vitamin D receptor (VDR) expression in intestinal tissues and corresponding expression level analysis (scale bar = 200 μm; n = 6 in each group). **(C)** Western blot analysis and densitometric quantification of VDR, activating transcription factor 4 (ATF4), and C/EBP homologous protein (CHOP) expression levels (n = 6 in each group). Statistical analysis was performed using one-way ANOVA followed by Tukey’s *post hoc* test. All data are presented as mean ± SD. *, P values <0.05; **, *P* values <0.01; ****, *P* values <0.0001.

On transmission electron microscopy, I/R was seen to cause swelling and rupture of the endoplasmic reticulum in intestinal mucosal epithelial cells, which was alleviated by pretreatment with paricalcitol. After pretreatment, only mild swelling of the endoplasmic reticulum was observed (Figure 2A). IHC analysis demonstrated that paricalcitol pretreatment restored the intestinal VDR protein expression level, which was reduced by I/R injury, to normal levels (Figure 2B). Western blotting similarly demonstrated that paricalcitol mitigated the I/R-induced downregulation of VDR expression and inhibited the upregulation of ATF4 and CHOP (Figure 2C).

### VDR-KO aggravated intestinal I/R injury and ERS

**FIGURE 3:**
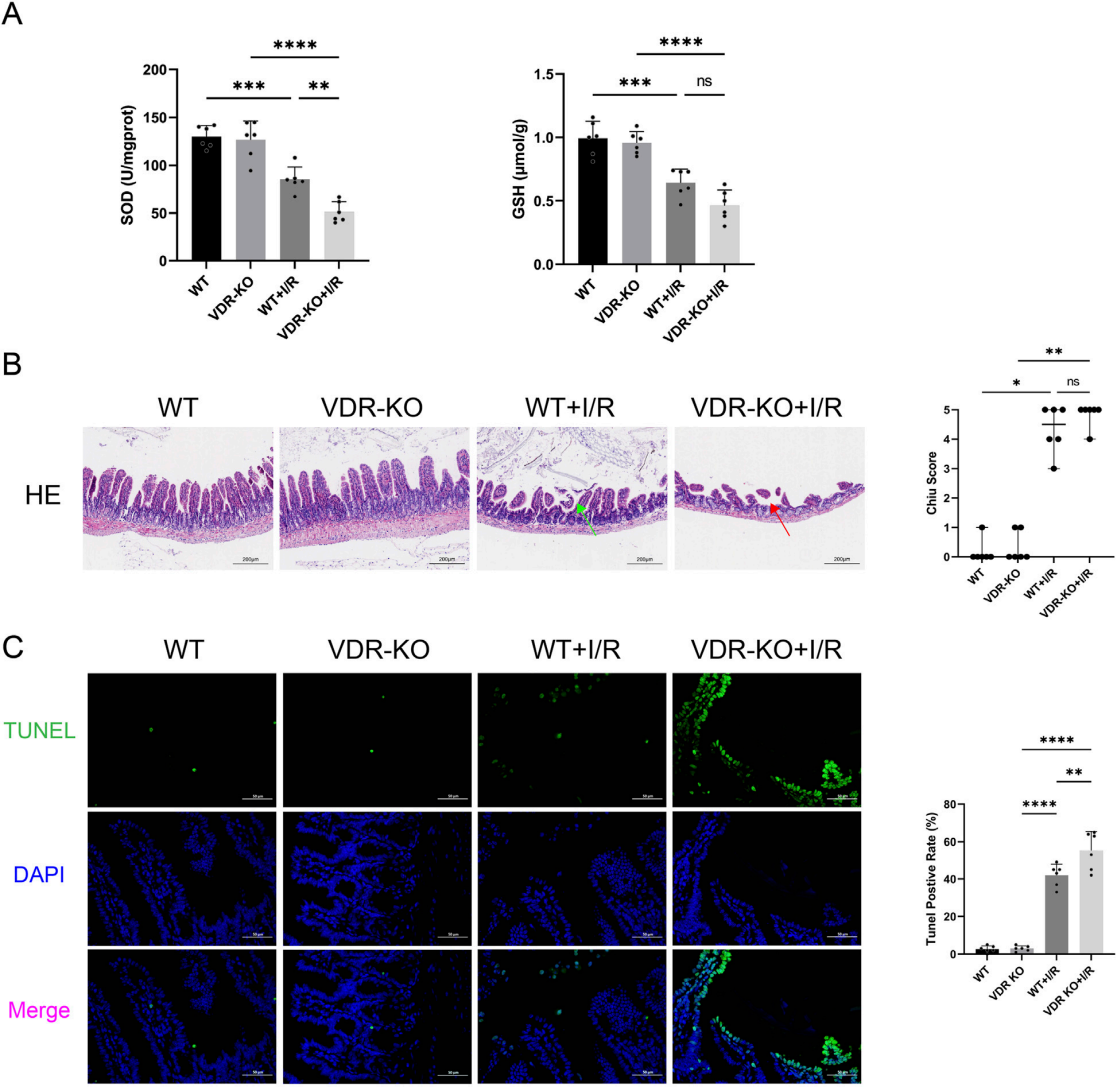
Vitamin D receptor (VDR) knockout (KO) exacerbates intestinal ischemia reperfusion (I/R) injury and cell apoptosis **(A)** Levels of superoxide dismutase (SOD) and glutathione (GSH) in intestinal tissues of VDR-KO mice (n = 6 in each group). **(B)** Representative images of intestinal hematoxylin and eosin , (H&E) staining results and Chiu scores for VDR-KO mice. Red arrow represents severe villous denudation, green arrow represents mild denudation of the villous tips (scale bar = 200 μm; n = 6 in each group). **(C)** Representative TUNEL staining (green) and nuclear staining (blue) results and apoptosis analysis in intestinal tissues of VDR-KO mice (scale bar = 50 μm; n = 6 in each group). Statistical analysis was performed using one-way ANOVA followed by Tukey’s *post hoc* test for parametric data and the Kruskal–Wallis test followed by Dunn’s *post hoc* test for non-parametric data. Parametric data are presented as mean ± SD, while non-parametric data are presented as median (min-max). *, P values <0.05; **, *P* values <0.01; ***, *P* values <0.001; ****, *P* values <0.0001.

We found that in VDR-KO mice, I/R injury reduced intestinal SOD activity to only 40% of the original level, while in WT mice, the reduction was to 65%. The absence of VDR significantly exacerbated the decrease in SOD activity caused by I/R injury (*P* < 0.01) (Figure 3A). H&E staining indicated that intestinal damage caused by I/R could be more severe in VDR-KO mice than in WT mice, although the difference was not statistically significant (Figure 3B). TUNEL analysis revealed that the apoptosis rate of intestinal epithelial cells in the VDR-KO + I/R group was elevated by 31% compared to the WT + I/R group (P < 0.01) (Figure 3C).

**FIGURE 4:**
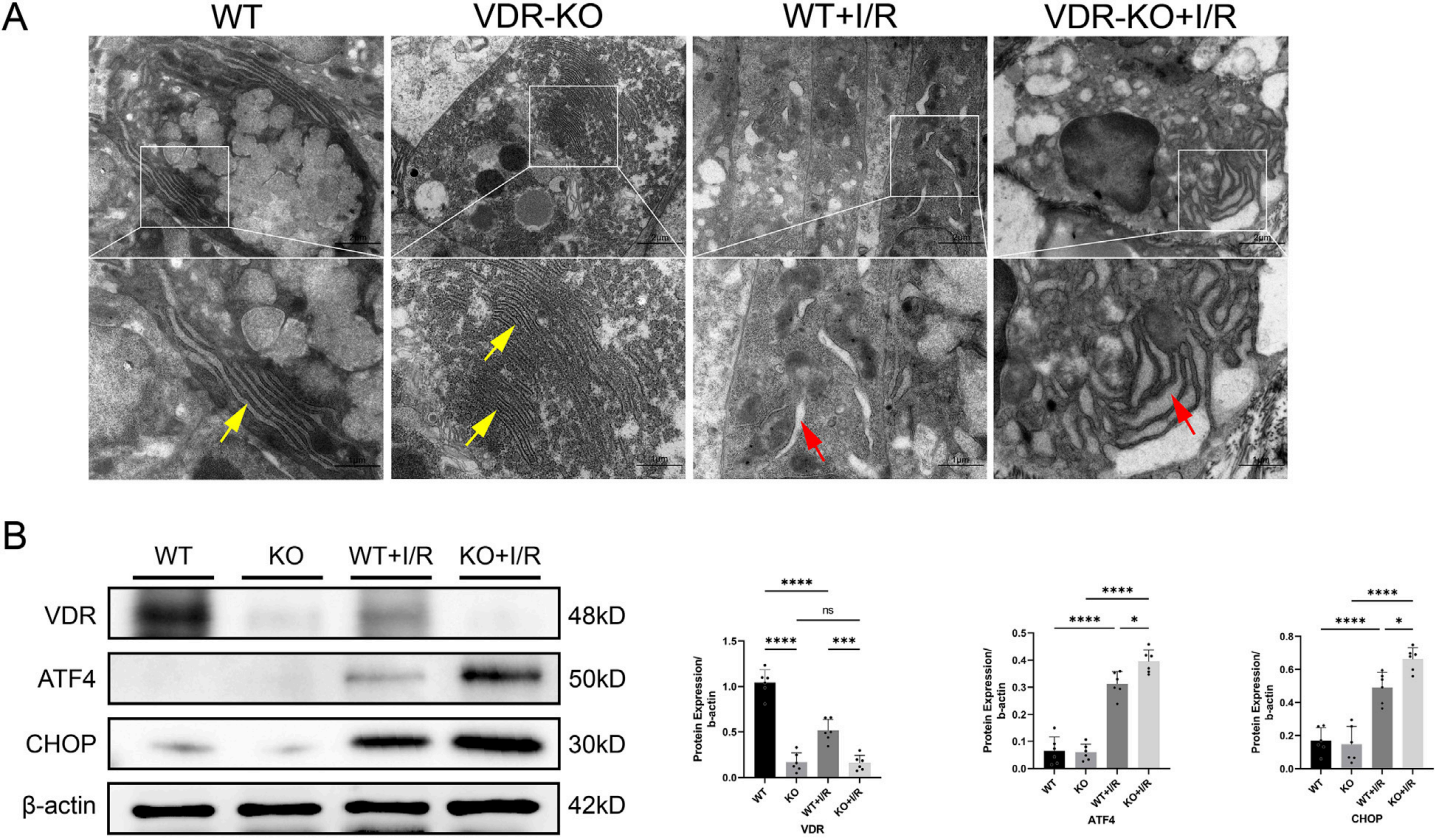
Vitamin D receptor (VDR) knockout (KO) exacerbates intestinal ischemia-reperfusion (I/R) injury by inducing endoplasmic reticulum stress (ERS) **(A)** Representative transmission electron microscopy images showing endoplasmic reticulum damage in intestinal epithelial cells of VDR-KO mice. Yellow arrows represent normal endoplasmic reticulum, red arrows represent endoplasmic reticulum changes (scale bar = 2 or 1 μm; n = 3 in each group). **(B)** Western blot analysis and densitometric quantification of VDR, activating transcription factor 4 (ATF4), and C/EBP homologous protein (CHOP) expression levels (n = 6 in each group). Statistical analysis was performed using one-way ANOVA followed by Tukey’s *post hoc* test. All data are presented as mean ± SD. *, P values <0.05; **, *P* values <0.01; ***, *P* values <0.001; ****, *P* values <0.0001.

Electron microscopy analysis showed that, compared to the WT + I/R group, the VDR-KO + I/R group exhibited more pronounced endoplasmic reticulum swelling, rupture, and loss of normal structure (Figure 4A). This suggests that VDR knockout may exacerbate intestinal I/R injury by intensifying I/R-induced ERS. Western blotting results indicated that VDR deficiency increased the accumulation of ATF4 and CHOP proteins (Figure 4B).

### Paricalcitol mitigated H/R injury in IEC-6 cells

**FIGURE 5:**
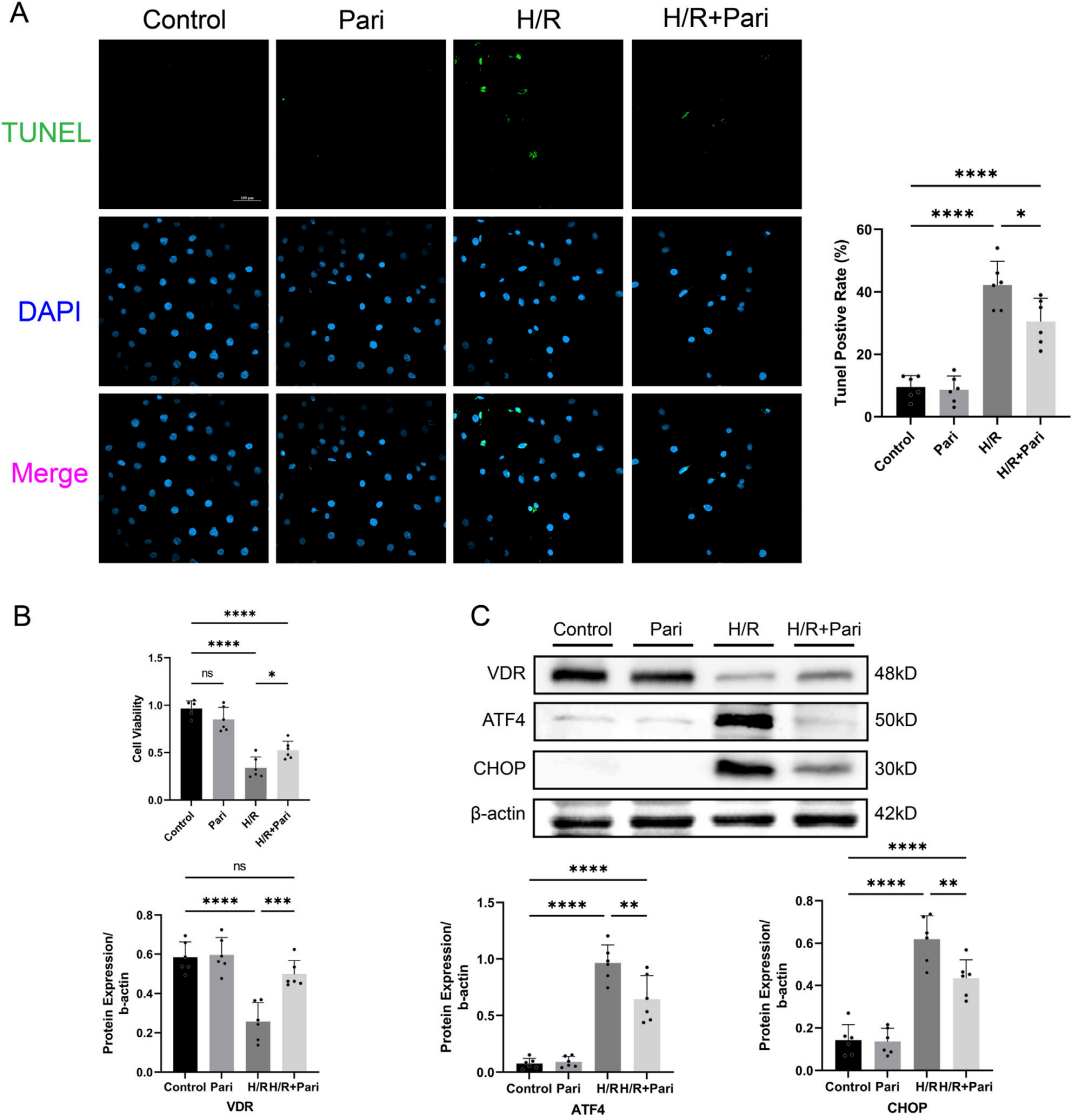
Paricalcitol mitigates hypoxia-reoxygenation (H/R) injury in IEC-6 cells **(A)** Representative TUNEL staining (green) and nuclear staining (blue) results and apoptosis analysis of IEC-6 cells (scale bar = 100 μm; n = 6 in each group). **(B)** Differences in IEC-6 cell viability among groups assessed using the cell counting kit-8 assay (n = 6 in each group). **(C)** Western blot analysis and densitometric quantification of vitamin D receptor (VDR), activating transcription factor 4 (ATF4), and C/EBP homologous protein (CHOP) expression levels in IEC-6 cells (n = 6 in each group). Statistical analysis was performed using one-way ANOVA followed by Tukey’s *post hoc* test. All data are presented as mean ± SD. *, P values <0.05; **, *P* values <0.01; ***, *P* values <0.001; ****, *P* values <0.0001.

TUNEL assay demonstrated that the rate of apoptotic cells in the paricalcitol-treated H/R group was reduced by 28% compared to the H/R group without paricalcitol treatment (*P* < 0.05) (Figure 5A). CCK-8 assay demonstrated that H/R injury reduced the viability of IEC-6 cells to 35% of the control group (*P* < 0.0001). However, with paricalcitol treatment, cell viability was maintained at 54% of the control level, indicating that paricalcitol significantly mitigated the H/R-induced decline in cell viability (*P* < 0.05) (Figure 5B), and Western blotting indicated that H/R led to downregulation of VDR expression and upregulation of ATF4 and CHOP in IEC-6 cells, which were reversed by paricalcitol pretreatment (Figure 5C).

### Silencing VDR or ATF4 abolished the protective effect of paricalcitol in IEC-6 cells

**FIGURE 6:**
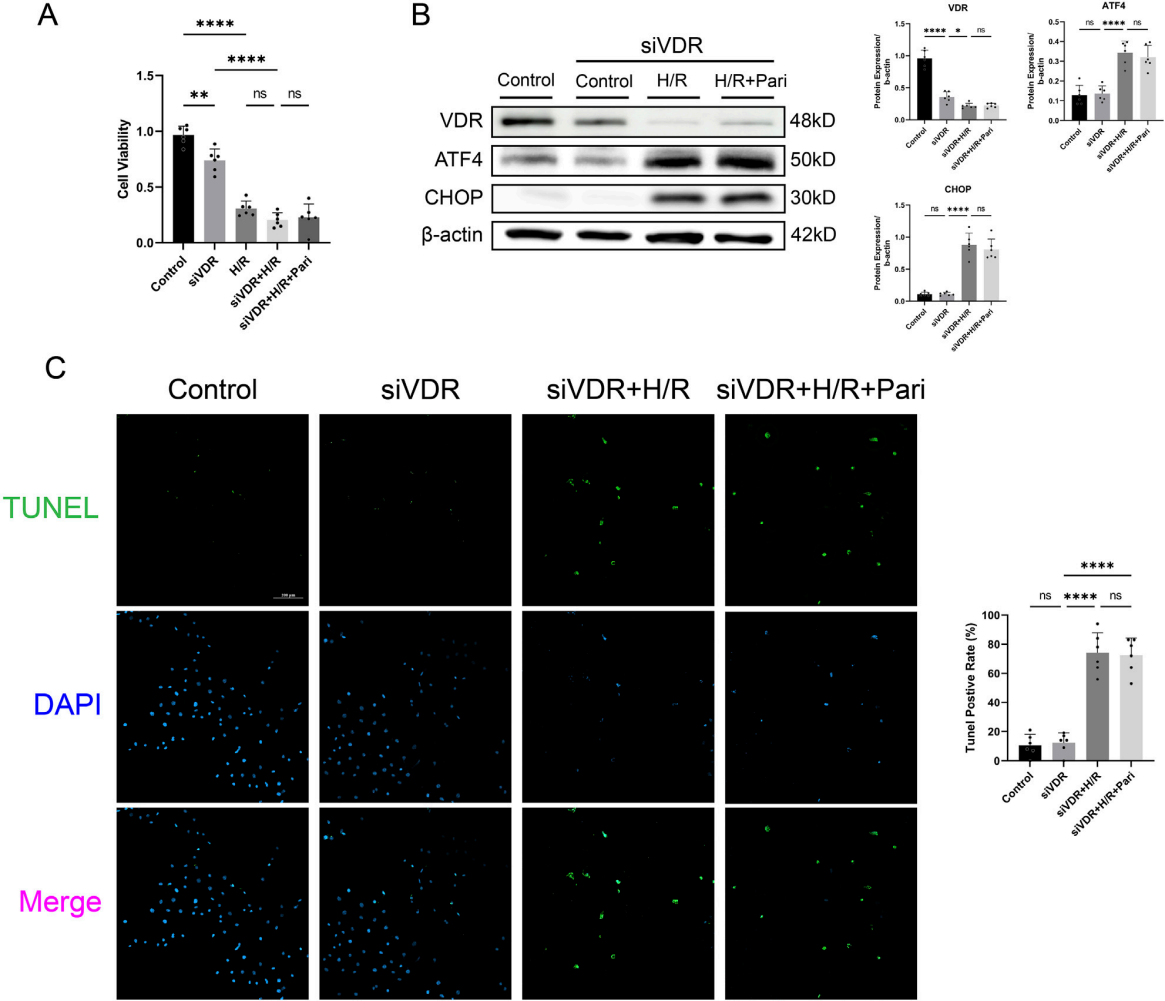
Silencing vitamin D receptor (VDR) abolished the protective effect of paricalcitol in IEC-6 cells **(A)**The effect of siVDR treatment on cell viability assessed using cell counting kit-8 assay in IEC-6 cells (n = 6 in each group). **(B)** Western blot analysis and densitometric quantification of VDR, ATF4, and CHOP expression levels in IEC-6 cells after siVDR treatment (n = 6 in each group). **(C)** Representative TUNEL staining (green) and nuclear staining (blue) results for IEC-6 cells after siVDR treatment (scale bar = 200 μm; n = 6 in each group). Statistical analysis was performed using one-way ANOVA followed by Tukey’s *post hoc* test. All data are presented as mean ± SD. *, P values <0.05; **, P values <0.01; ***, P values <0.001; ****, P values <0.0001.

CCK-8 assay results indicated that after H/R injury, cell viability in the VDR-silenced and non-silenced groups decreased by 2.3-fold and 4.8-fold, respectively, compared to the control group (*P* < 0.0001). However, there was no significant difference between the H/R group and siVDR + H/R groups. In the siVDR + H/R group, subsequent treatment with paricalcitol did not restore the H/R-induced decrease of cell viability (Figure 6A). This contrasts with the protective effect observed with paricalcitol treatment in Figure 5B. Western blotting results showed that after siVDR treatment, ATF4 and CHOP expression remained similar regardless of paricalcitol treatment, indicating that silencing VDR abolished the effect of paricalcitol (Figure 6B). TUNEL staining yielded similar results, showing that silence VDR abolished the protective effect of paricalcitol against cell apoptosis (Figure 6C).

**FIGURE 7:**
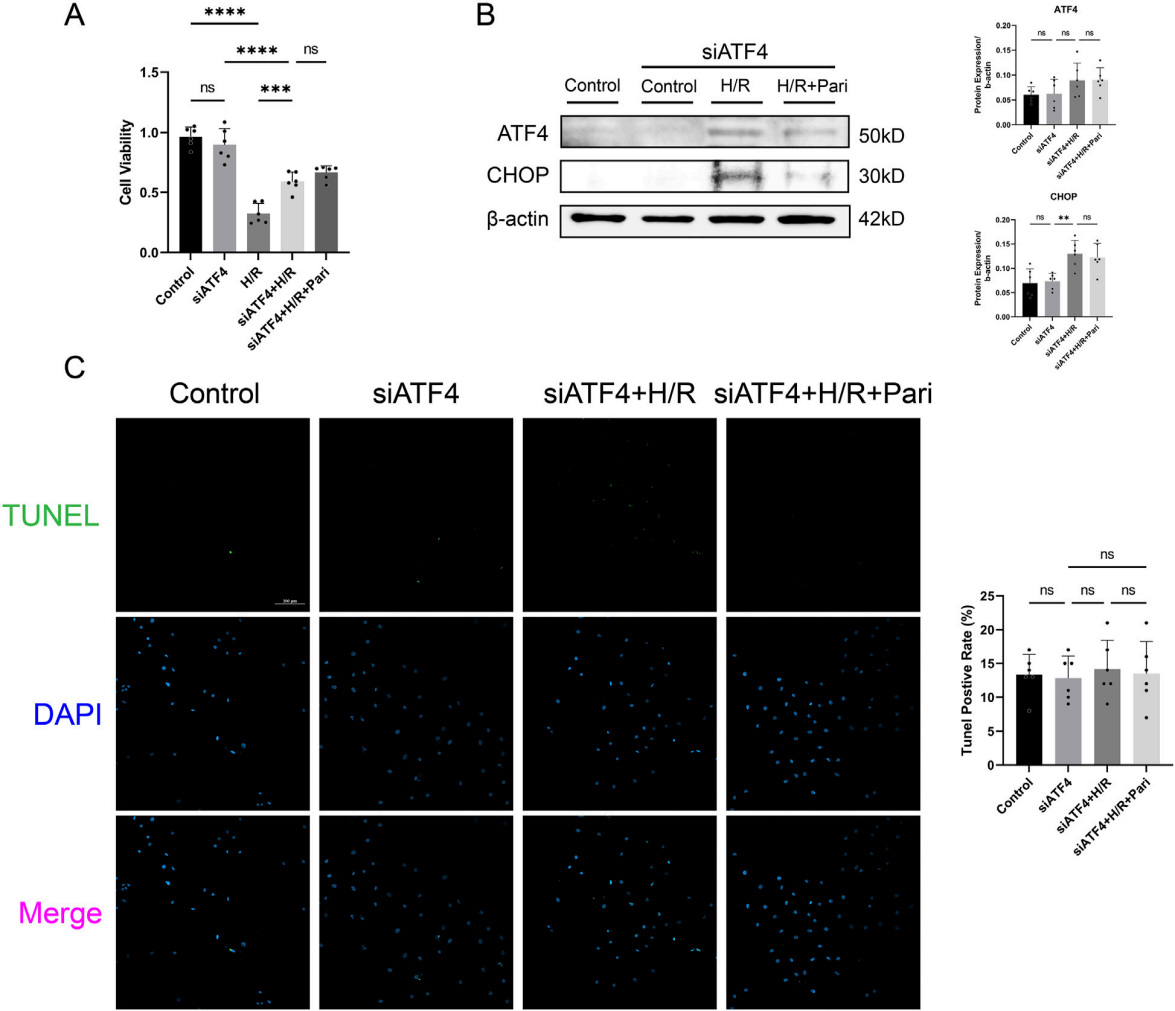
Silencing activating transcription factor 4 (ATF4) abolished the protective effect of paricalcitol in IEC-6 cells **(A)** The effect of siATF4 treatment using cell counting kit-8 assay on cell viability in IEC-6 cells (n = 6 in each group). **(B)** Western blot analysis and densitometric quantification of ATF4 and CHOP expression levels in IEC-6 cells after siATF4 treatment (n = 6 in each group). **(C)** Representative TUNEL staining (green) and nuclear staining (blue) results for IEC-6 cells after siATF4 treatment assay (scale bar = 200 μm; n = 6 in each group). Statistical analysis was performed using one-way ANOVA followed by Tukey’s *post hoc* test. All data are presented as mean ± SD. *, P values <0.05; **, *P* values <0.01; ***, *P* values <0.001; ****, *P* values <0.0001.

CCK-8 assay showed that H/R treatment significantly reduced cell viability in both ATF4-silenced and non-silenced cells, by 3-fold and 1.6-fold, respectively, compared to the control group (*P* < 0.0001). Compared to the H/R group, cell viability in the siATF4 + H/R group increased by 1.8-fold, indicating that silencing ATF4 alleviated the reduction in cell viability caused by H/R injury (*P* < 0.001). However, paricalcitol treatment was unable to further enhance cell viability (Figure 7A). Western blotting results showed that after ATF4 silencing, H/R increased CHOP expression, while paricalcitol treatment did not significantly alter CHOP expression (Figure 7B). This suggests that the protective effect of paricalcitol was suppressed after ATF4 silencing. Likewise, TUNEL staining showed that after ATF4 silencing, paricalcitol treatment did not significantly affect cell apoptosis levels (Figure 7C).

## Discussion

**FIGURE 8:**
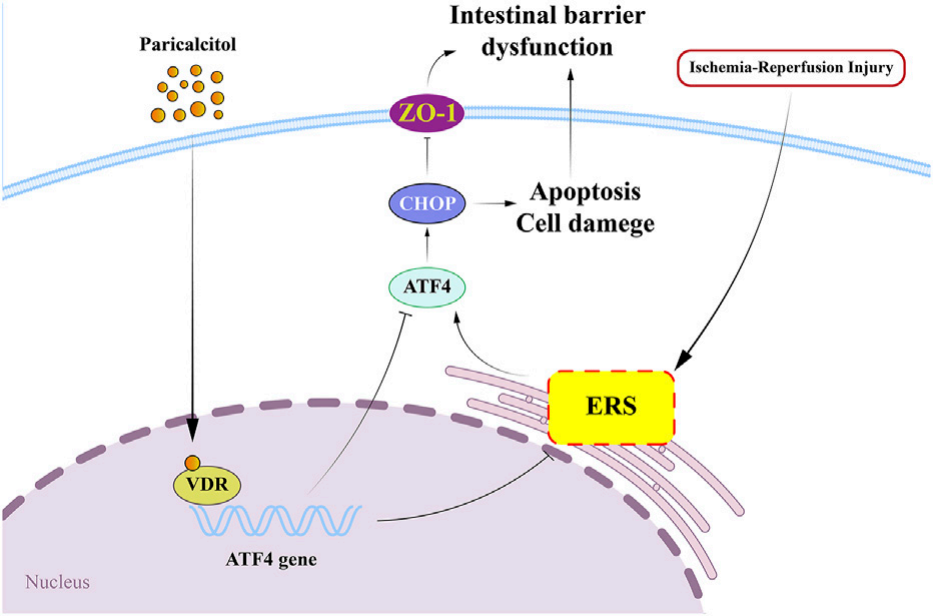
Paricalcitol alleviates intestinal ischemia-reperfusion injury by activating vitamin D receptor (VDR) and downregulated transcription factor 4 (ATF4) - C/EBP homologous protein (CHOP) expression VDR, vitamin D receptor; ERS, endoplasmic reticulum stress; ATF4, downregulated transcription factor 4; CHOP, C/EBP homologous protein; ZO-1, zonula occludens-1.

In this study, we found that paricalcitol alleviates intestinal I/R injury by activating VDR signaling. Paricalcitol markedly downregulated ATF4 and CHOP expression, thereby mitigating ERS, apoptosis, and intestinal barrier damage caused by I/R injury (Figure 8). Conversely, VDR knockout worsened intestinal I/R injury and increased ATF4 and CHOP levels. *In vivo* experimental results demonstrated that paricalcitol significantly alleviated H/R-induced injury in IEC-6 cells, activated VDR, and reduced ATF4 and CHOP protein expression. Notably, we observed that VDR silencing led to a marked decrease in cell viability and an increase in apoptosis, regardless of paricalcitol treatment. This suggests that the protective effect of paricalcitol against H/R injury is mediated through VDR activation. Furthermore, ATF4 silencing significantly improved cell viability following H/R injury, similar to the effect of paricalcitol. However, paricalcitol treatment did not further enhance cell viability in ATF4-silenced cells, indicating that ATF4 suppression alone is sufficient to mitigate H/R injury. Despite this, ATF4 silencing did not result in significant changes in apoptosis levels or CHOP protein expression after H/R injury. Given that ATF4 is not the sole upstream regulator of CHOP, their interaction may be more complex, warranting further investigation.

Previous studies have shown that VDR, as a transcription factor, can directly bind to the ATF4 promoter, inhibiting its protein expression (He et al., 2024). This aligns with our findings, as we confirmed the regulatory effect of VDR on downstream ATF4 using VDR-KO mice and *in vitro* transfection of siVDR. ATF4, as a key mediator of ERS, plays a crucial role in ERS-associated apoptosis (Ren et al., 2021). Research has shown that activation of the ATF4-CHOP pathway promotes apoptosis in porcine intestinal epithelial cells (Li et al., 2024). Conversely, blocking this pathway has been shown to reduce ERS and mitigate dextran sulfate sodium–induced colitis (Fan et al., 2024), and silencing ATF4 *in vitro* has been found to substantially opposed H/R-induced ERS and protected cells from H/R damage (Liu et al., 2024). Our study further expands on the role of the ATF4-CHOP pathway in intestinal I/R injury and suggests a potential therapeutic approach for this condition.

Studies on inflammatory bowel disease have shown that moderate ERS helps maintain intestinal homeostasis, protecting the normal function of the mucosal epithelium. However, excessive ERS activation can lead to inflammation, epithelial cell apoptosis, and disruption of the intestinal mucosal barrier (Kaur and Debnath, 2015; Qiao et al., 2021). Currently, ERS is understood to be initiated by three endoplasmic reticulum transmembrane sensors: inositol-requiring enzyme 1 (IRE1), protein kinase R-like endoplasmic reticulum kinase (PERK), and activating transcription factor 6 (ATF6) (Wu et al., 2024). Binding immunoglobulin protein (BiP) interactions with nucleotides primarily mediate these processes (Pobre et al., 2019). Under ERS, BiP dissociates to activate IRE1, PERK, or ATF6, initiating a cascade of ERS and downstream signaling (Ma et al., 2017). In the PERK pathway, PERK oligomerization and phosphorylation activate eukaryotic initiation factor 2α (eIF2α), leading to ATF4 expression, which is induced by eIF2α phosphorylation (Vattem and Wek, 2004). This increases CHOP expression, ultimately inducing apoptosis (Rozpedek et al., 2016). Our study expands on this understanding, demonstrating that VDR activation can directly inhibit ATF4-CHOP expression. This suggests that the ATF4-CHOP pathway may be regulated by both PERK and VDR and that normal activation of VDR is essential in maintaining endoplasmic reticulum homeostasis.

This study had some limitations. First, we only investigated protein expression changes after paricalcitol intervention without measuring mRNA levels or examining transcriptional changes. Second, although previous research has shown that VDR can bind to the ATF4 promoter and repress its transcription, we did not confirm this in our study; further studies are needed to assess the molecular interactions between VDR and ATF4. Third, in our experiments silencing VDR and ATF4, we did not include an H/R group with non-silenced cells, therefore, we cannot determine the specific effects of VDR and ATF4 silencing on H/R injury. Fourth, we only investigated the protective effects of paricalcitol pre-treatment on I/R but did not examine its potential effects as a post-treatment. Although using paricalcitol after I/R would be more clinically relevant, we have not yet explored this aspect. Last, the mechanisms of intestinal I/R injury are complex and multisystemic. We restricted our focus to the effects of paricalcitol on the intestines and intestinal epithelial cells, overlooking potential interactions among different organs and cell types. Therefore, the full role of paricalcitol in intestinal I/R injury warrants further investigation.

In conclusion, our work demonstrates that the ability of paricalcitol to activate VDR offers protection against intestinal I/R injury by inhibiting ERS, primarily through the ATF4-CHOP pathway. The role of paricalcitol and the ATF4-CHOP pathway in intestinal I/R injury had not been previously reported. Our study highlights a potential link between these factors and ERS, providing new insights into the mechanisms of intestinal injury and potential therapeutic approaches.

## Data Availability

The original contributions presented in the study are included in the article/supplementary material, further inquiries can be directed to the corresponding authors.
