# Corrigendum: Paricalcitol alleviates intestinal ischemia-reperfusion injury via inhibition of the ATF4-CHOP pathway

**DOI:** 10.3389/fphar.2025.1632205

**Published:** 2025-06-25

**Authors:** Jiawei Zhang, Tingting Liu, Tongqing Xue, Zhongzhi Jia

**Affiliations:** ^1^ Department of Interventional and Vascular Surgery, The Third Affiliated Hospital of Nanjing Medical University (Changzhou Second People’s Hospital), Changzhou, China; ^2^ Graduate College of Dalian Medical University, Dalian, China; ^3^ Department of Interventional Radiology, Huaian Hospital of Huai’an City (Huaian Cancer Hospital), Huai’an, China

**Keywords:** intestinal, ischemia reperfusion injury, paricalcitol, ATF4, CHOP, VDR (vitamin D receptor)

In the published article, there were errors in Figures 5C, 6C. Due to Authors’ oversight during figure preparation, the ATF4 and β-actin image that was originally intended for **Figure 2C** was mistakenly placed again in Figure 5C. Additionally, in Figure 6C, the TUNEL and DAPI staining images for the siATF4 group were incorrectly inserted. The authors also replaced the ATF4 and β-actin images in Figure 6, as the originally selected images were not sufficiently representative of the observed trends. The corrected Figures 5, 6 and its caption appear below.

**FIGURE 5:**
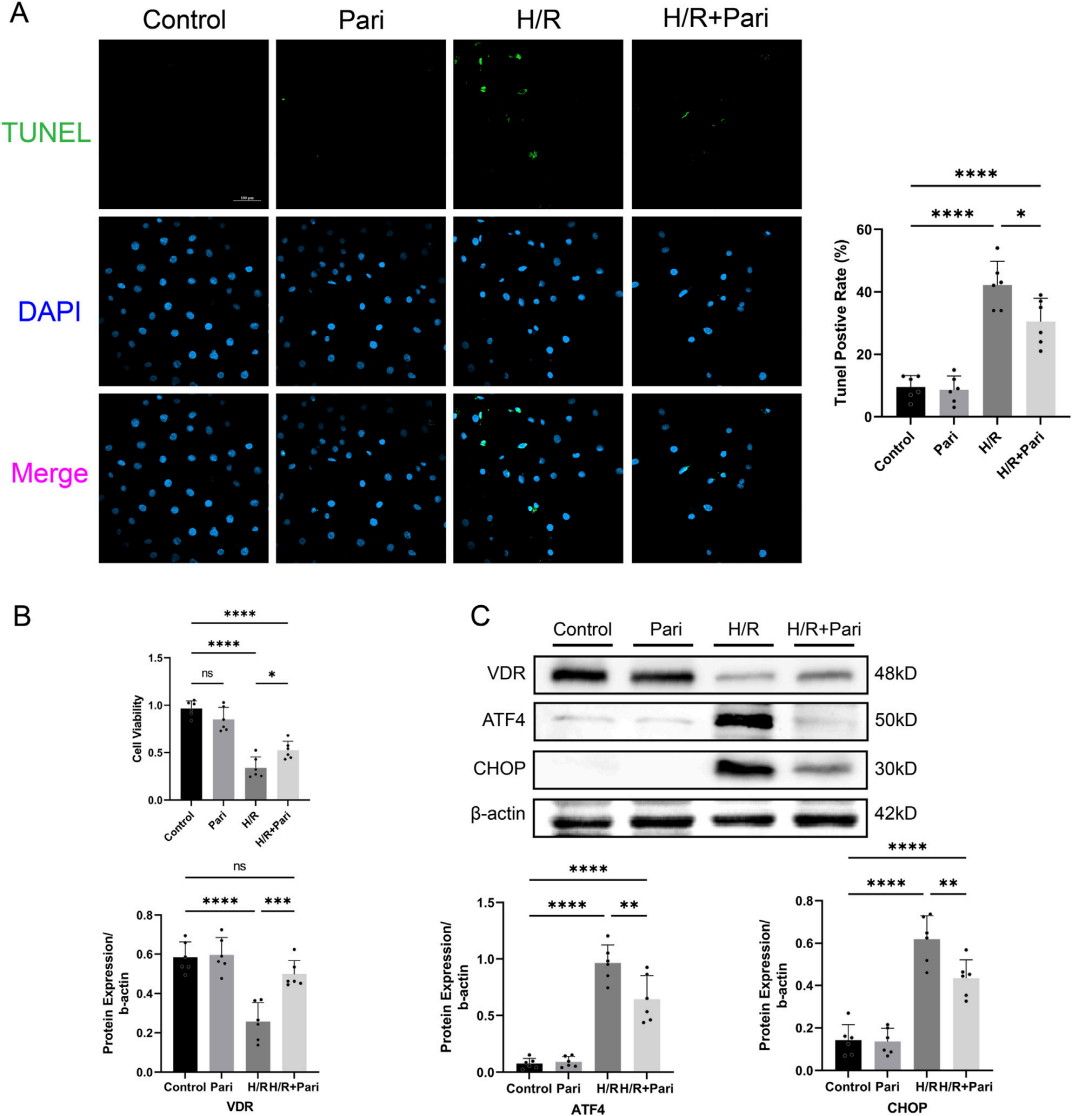
Paricalcitol mitigates hypoxia-reoxygenation (H/R) injury in IEC-6 cells **(A)** Representative TUNEL staining (green) and nuclear staining (blue) results and apoptosis analysis of IEC-6 cells (scale bar = 100 μm; n = 6 in each group). **(B)** Differences in IEC-6 cell viability among groups assessed using the cell counting kit-8 assay (n = 6 in each group). **(C)** Western blot analysis and densitometric quantification of vitamin D receptor (VDR), activating transcription factor 4 (ATF4), and C/EBP homologous protein (CHOP) expression levels in IEC-6 cells (n = 6 in each group). Statistical analysis was performed using one-way ANOVA followed by Tukey’s *post hoc* test. All data are presented as mean ± SD. *, P values <0.05; **, *P* values <0.01; ***, *P* values <0.001; ****, *P* values <0.0001.

**FIGURE 6:**
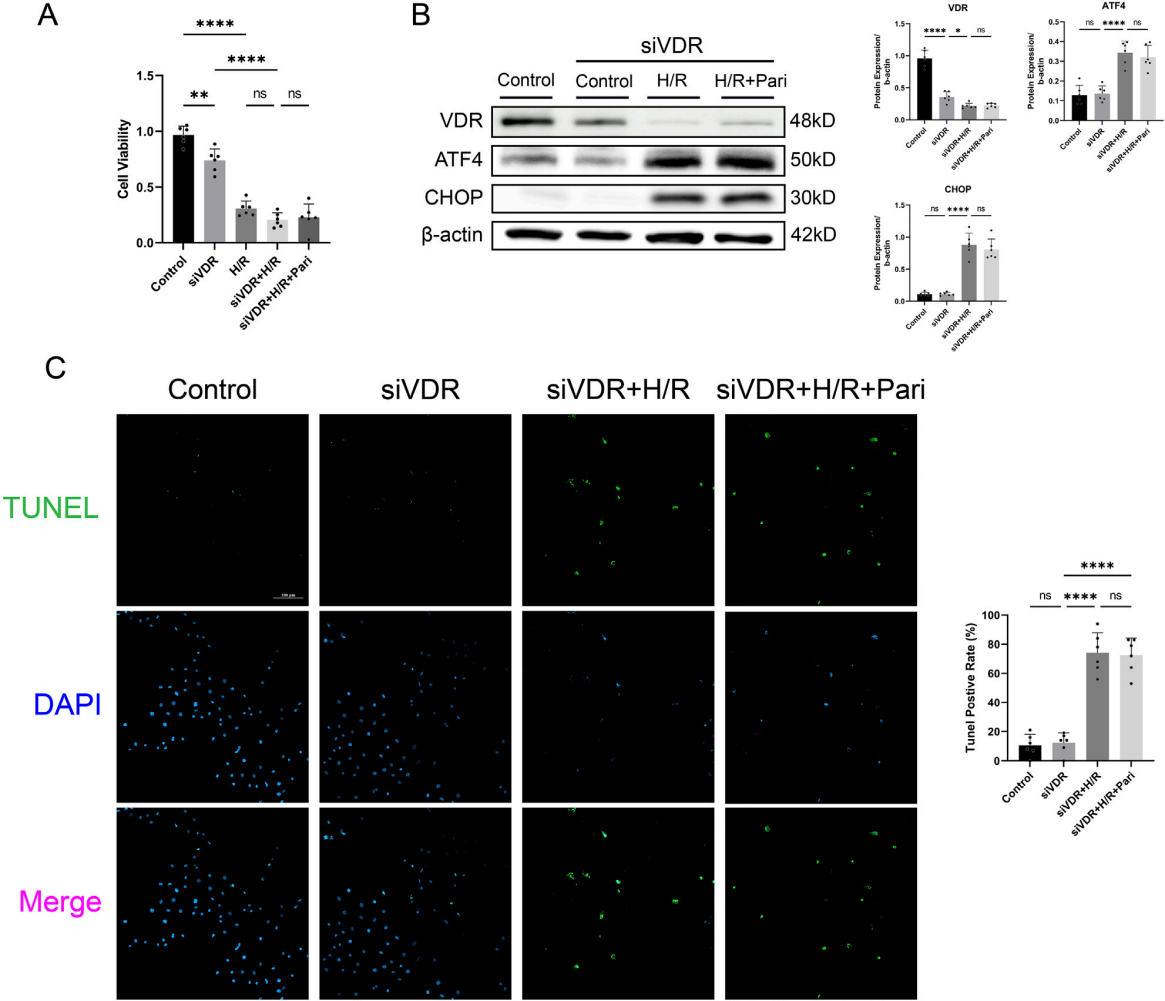
Silencing vitamin D receptor (VDR) abolished the protective effect of paricalcitol in IEC-6 cells **(A)**The effect of siVDR treatment on cell viability assessed using cell counting kit-8 assay in IEC-6 cells (n = 6 in each group). **(B)** Western blot analysis and densitometric quantification of VDR, ATF4, and CHOP expression levels in IEC-6 cells after siVDR treatment (n = 6 in each group). **(C)** Representative TUNEL staining (green) and nuclear staining (blue) results for IEC-6 cells after siVDR treatment (scale bar = 200 μm; n = 6 in each group). Statistical analysis was performed using one-way ANOVA followed by Tukey’s *post hoc* test. All data are presented as mean ± SD. *, P values <0.05; **, P values <0.01; ***, P values <0.001; ****, P values <0.0001.

The authors apologize for this error and state that this does not change the scientific conclusions of the article in any way. The original article has been updated.

